# Synergistic Anti Leukemia Effect of a Novel Hsp90 and a Pan Cyclin Dependent Kinase Inhibitors

**DOI:** 10.3390/molecules25092220

**Published:** 2020-05-08

**Authors:** Ashraf N. Abdalla, Mohamed E. Abdallah, Akhmed Aslam, Ammar Bader, Antonio Vassallo, Nunziatina De Tommasi, Waleed H. Malki, Ahmed M. Gouda, Mohammed H. Mukhtar, Mahmoud Zaki El-Readi, Hamad M. Alkahtani, Alaa A.-M. Abdel-Aziz, Adel S. El-Azab

**Affiliations:** 1 College of Pharmacy, Umm Al-Qura University, Makkah 21955, Saudi Arabia; ambader@uqu.edu.sa (A.B.); whmalki@uqu.edu.sa (W.H.M.); 2 Faculty of Medicine, Umm Al-Qura University, Makkah 21955, Saudi Arabia; mezubier@uqu.edu.sa (M.E.A.); mhmukhtar@uqu.edu.sa (M.H.M.); mzreadi@uqu.edu.sa (M.Z.E.-R.); 3 Faculty of Applied Medical Sciences, Umm Al-Qura University, Makkah 21955, Saudi Arabia; maaslam@uqu.edu.sa; 4 Dipartimento di Scienze, Università Degli Studi della Basilicata, 85100 Potenza, Italy; antonio.vassallo@unibas.it; 5 Dipartimento di Farmacia, Università Degli Studi di Salerno, 84084 Fisciano (SA), Italy; detommasi@unisa.it; 6 Faculty of Pharmacy, Beni-Suef University, Beni-Suef 62514, Egypt; ahmed.gouda@pharm.bsu.edu.eg; 7 Faculty of Pharmacy, Al-Azhar University, Assiut, 71524, Egypt; 8 College of Pharmacy, King Saud University, Riyadh 11451, Saudi Arabia; ahamad@ksu.edu.sa (H.M.A.); almoenes@ksu.edu.sa (A.A.-M.A.-A.); adelazab@ksu.edu.sa (A.S.E.-A.)

**Keywords:** acute myeloid leukemia (AML), Hsp90, cyclin-dependent kinases (CDK), dinaciclib

## Abstract

Acute myeloid leukemia (AML) is among the top four malignancies in Saudi nationals, and it is the top leukemia subtype worldwide. Resistance to available AML drugs requires the identification of new targets and agents. Hsp90 is one of the emerging important targets in AML, which has a central role in the regulation of apoptosis and cell proliferation through client proteins including the growth factor receptors and cyclin dependent kinases. The objective of the first part of this study is to investigate the putative Hsp90 inhibition activity of three novel previously synthesized quinazolines, which showed HL60 cytotoxicity and VEGFR2 and EGFR kinases inhibition activities. Using surface plasmon resonance, compound 1 (HAA_2020_) showed better Hsp90 inhibition compared to 17-AAG, and a docking study revealed that it fits nicely into the ATPase site. The objective of the second part is to maximize the anti-leukemic activity of HAA_2020_, which was combined with each of the eleven standard inhibitors. The best resulting synergistic effect in HL60 cells was with the pan cyclin-dependent kinases (CDK) inhibitor dinaciclib, using an MTT assay. Furthermore, the inhibiting effect of the Hsp90α gene by the combination of HAA_2020_ and dinaciclib was associated with increased caspase-7 and TNF-α, leading to apoptosis in HL60 cells. In addition, the combination upregulated p27 simultaneously with the inhibition of cyclinD3 and CDK2, leading to abolished HL60 proliferation and survival. The actions of HAA_2020_ propagated the apoptotic and cell cycle control properties of dinaciclib, showing the importance of co-targeting Hsp90 and CDK, which could lead to the better management of leukemia.

## 1. Introduction

According to the Saudi Cancer Registry (2014), leukemia ranked second and sixth among the top malignancies for Saudi males and females, respectively, while it is the top malignancy for both Saudi sexes below 14 years of age [1]. Moreover, acute myeloid leukemia (AML) ranked fourth, with 11.9% of the total leukemia cases in both Saudi sexes during 1999–2013. The most common subtype of AML in Saudis is M1, with myeloperoxidase, CD13, CD33 and CD117 as the most common reported antigens [2,3]. Globally, AML is considered the top leukemia subtype, with a higher incidence in elderly patients [4].

Reviewing some of the effective targets, FLT3 is regarded as one of the most important mutations in AML, which is prevalent in over a third of its cases [5]. Sunitinib, sorafenib, lestaurtinib and midostaurin are considered the first generation of FLT3 inhibitors, as they have shown a broader effect on kinases VEGFR, PDGFR, KIT, JAK2 and RAS/RAF, beside FLT3 as a main target. Additionally, a number of recently FDA-approved, more FLT3-specific AML inhibitors have offered more promising treatment possibilities including quizartinib, crenolanib and gilteritinib [6]. Moreover, new combinations have shown a prolonged response and lower resistance rate, such as the combination of either midostaurin or glasdegib with cytarabine [4,6]. On another direction of targeting oncogenic mutations, enasidenib and ivosidenib are considered the prototypes of the isocitrate dehydrogenase (IDH) 1 and 2 inhibitors, which may benefit 7–19% of AML patients, yet their clinical significance is pending running clinical trials [4]. However, despite the achievement of complete remission (CR) for some time by many of the established AML inhibitors, multiple AML drug resistance pathways enable a quick recurrence. These resistance pathways include the FLT3, IDH, Bcl2, MDM2, PD1, CD33, CD123, WT1 and RAS family gene alterations [7,8]. Thus, resistance remains a major hurdle, which necessitates the continuation of both new target and drug discoveries [9]. 

One of the emerging targets for cancer therapeutics are the cyclin-dependent kinases (CDKs), which are important cell cycle regulatory proteins. Many CDK inhibitors have been recently approved for AML [10]. For example, the CDK9 inhibitor alvocidib showed encouraging clinical results for refractory AML through the MCL-1-dependent pathway, while several CDK 4/6 inhibitors are under development to downregulate the FLT3 and KMT2A genes in AML. Additionally, dinaciclib (pan CDK 1, 2, 5 and 9 inhibitor), which showed good tolerability, is being tested as a monotherapy or in combination with venetoclax (Bcl-2 inhibitor) in a relapsed and refractory AML clinical trial phase 1 [11]. Specifically, in HL60 cells, dinaciclib at 4–16 nM (2 h) increases the sub-G_1_ cell cycle phase, and induces high apoptosis (75% of total cells) [12].

Another attractive emerging target is the Hsp90, which has a central role in the regulation of survival, cell proliferation and apoptosis [13,14]. Cancer can be dependent on many Hsp90 clients like HER2 and EGFR [15]. It was found that the decrease in Hsp90 leads to the activation of caspases, and G_1_ arrest in leukemia cells [16,17,18]. Moreover, VEGF in HL60 cells were found to increase the expression of Hsp90, leading to cell survival, upregulation of VEGFR and downregulation of the apoptotic machinery [19].

Several Hsp90 inhibitors of natural or synthetic origins displayed potent anticancer activities. As an example, Y306zh exhibited anti-pancreatic cancer cell growth activity through the disruption of the p23, MAPK and Akt signalling pathways. It also distorted ATP binding to Hsp90, and downregulated the Hsp90 client proteins CDK4, Akt and EGFR [20]. Additionally, Hsp90 and surviving antagonist shepherdin induced apoptosis in HL60, K562, U937 and THP-1 patient-derived blast cells [21]. In another report, resistance in melanoma patients was discovered following the use of the combination of the Hsp90 inhibitor XL888 with the BRAF inhibitor vemurafenib, but the use of dinaciclib as a CDK2 inhibitor abolished the resistance of patients to the combination [22]. 

Moreover, the antibiotic and anticancer drug geldanamycin is known to degrade the Hsp90 substrate [23]. Further, its main derivative 17-*N*-allylamino-17-demethoxygeldanamycin (17-AAG) was found to inhibit Hsp90 and multiple tyrosine kinases (HER3, AXL, MET and EGFR), and increased p27 and caspases 3/7 in mesothelioma cell lines [24]. 17-AAG was also reported to increase the apoptotic effect of the CDK inhibitor flavopiridol in mantle cell lymphoma (MCL) and in the Kasumi-1 AML cell lines [25,26]. Additionally, the combination of the CDK inhibitor SCH727965 and the Hsp90 inhibitor NVP-AUY922 induced apoptosis and cell cycle disruption in sarcoma cells, while it spared the normal osteoblasts [27].

On the other hand, VEGF/VEGFR-1, -2 and -3 were found to provoke the proliferation of leukemia cells and increase tumorigenesis through the upregulation of hematopoietic growth factors. In addition, VEGF/VEGFR promote Hsp90 expression through binding to Bcl2, all of which builds up resistance against the apoptosis process. It was also found in AML patients that the increase in plasma VEGF levels was connected with a reduction in survival and remission rates and is a strong “independent” indicator of poor prognosis [28].

Recently, our collaborators in King Saud University-KSA synthetized a novel series of 2-[(3-(4-sulfamoylphenethyl)-4(3*H*)-quinazolinon-2-yl)thio]anilide derivatives. Compounds **1**–**5** showed cytotoxicity against the HL60, K562, MCF7 and HT29 cell lines, and displayed good selectivity towards the cancer cells compared with the MRC5 normal cells (structures of **1**–**5** are found in Supplementary Material Table S1). Compounds **1**, **4** and **5** displayed a potent inhibitory activity against the VEGFR2, EGFR and HER2 kinases, and their IC_50_ against HL60 cells were 1.7 µM, 1.5 µM and 0.4 µM, respectively. These three compounds showed better cytotoxicity in HL60 compared with orafenib: 3.14 µM [29].

These previous reports may explain the importance and relationships between CDKs, Hsp90, VEGFR2 and EGFR, which are prevalent in AML. Thus, the first objective of this exploratory drug discovery study is to test the putative Hsp90 inhibitory activity of **1**, **4** and **5**, and to select the best compound(s) for molecular docking using Hsp90α, as these compounds have previously shown an inhibition of the Hsp90 client proteins VEGFR2, EGFR and HER2. In the second step, a library of eleven inhibitors representing multiple leukemia targets, including CDKs, were tested in combination with the resulting Hsp90 inhibitor, for the possible maximization of its anti-leukemic activity. The main cancer hallmarks, cell cycle and apoptosis, were also investigated to explore the mechanistic features of the resulting compounds and their combinations. 

## 2. Results 

### 2.1. Surface Plasmon Resonance

**Figure 1:**
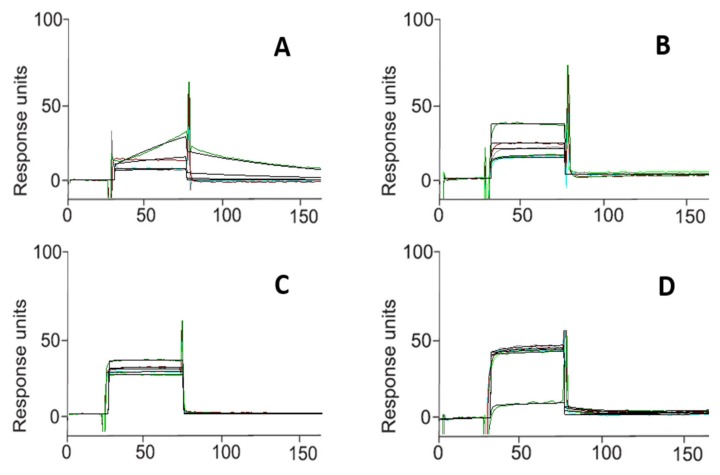
Surface plasmon resonance sensorgrams acquired for HAA_2020_ (**A**), **4** (**B**), **5** (**C**) interacting with Hsp90α and for the positive control 17-AAG (**D**). Each compound was injected onto an Hsp90α modified sensor chip at 6 (*n* = 3) different concentrations in the range 0.025–4.000 μM.

The interaction between each of the three compounds (**1**, **4**, and **5**) and Hsp90α was investigated in this study by a surface plasmon resonance (SPR)-based binding assay [30,31,32,33,34]. Only **1** (named HAA_2020_ in this study) interacted efficiently with the immobilized protein. As a result of fitting the relative sensorgrams to a single-site bimolecular interaction model, the thermodynamic parameters for the resulting complex formation were determined. This approach allowed the measurement of 51.0 ± 2.9 nM KD for the Hsp90α/HAA_2020_ complex. Interestingly, HAA_2020_ showed greater affinity towards the chaperone compared with that determined for 17-AAG (Table 1, Figure 1).

### 2.2. Molecular Docking Study

**Figure 2:**
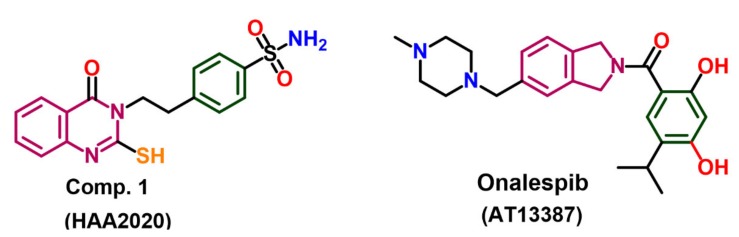
Chemical structure of HAA_2020_ and ATI-13387X.

Based on the result of the SPR (Table 1, Figure 1), the binding mode of HAA_2020_ into the ATPase site of Hsp90 was evaluated in a molecular docking study. The study was performed using AutoDock 4.2. Hsp90 protein with the co-crystallized onalespib (AT13387, Figure 2) as downloaded from Protein Data Bank (http://www.rcsb.org/pdb) [18].

**Figure 3:**
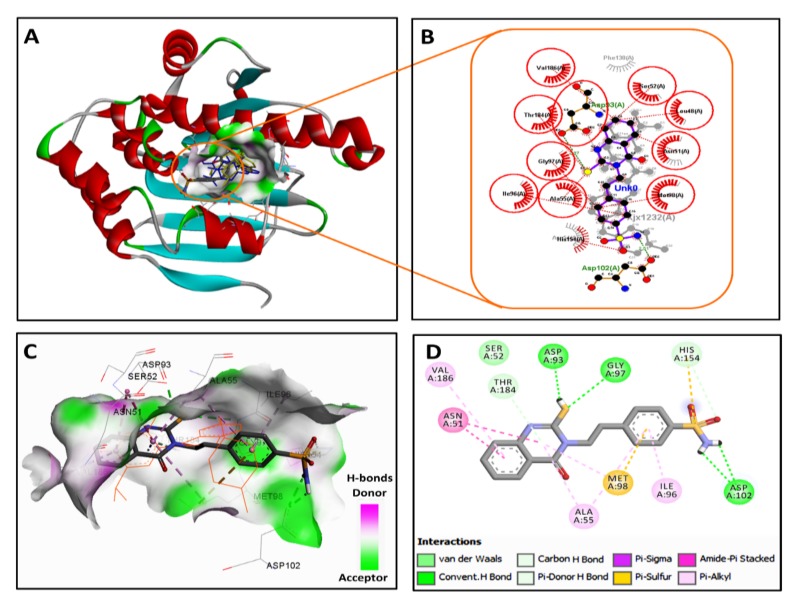
2/3D binding modes of onalespib and HAA_2020_ into the active site of Hsp90 (pdb code: 2XJX): (**A**) binding modes of the redocked onalespib (coloured in yellow), co-crystallized onalespib (coloured in blue) and HAA_2020_ (coloured by element) into Hsp90, (**B**) LigPlot view showing the superposition of HAA_2020_ and onalespib with two equivalent hydrogen bonds (shown as olive green dotted lines) with Asp93 and Asp102 and several equivalent hydrophobic interactions (shown as brick red dotted lines), (**C**) 3D binding mode of HAA_2020_ (shown as sticks coloured by element) overlaid with onalespib (shown as orange lines) into the binding site of Hsp90, receptor shown as the hydrogen bond surface, hydrogen atoms omitted for clarity, and (**D**) 2D binding mode of HAA_2020_ showing three conventional hydrogen bonds and different types of hydrophobic interactions.

The validation of the docking procedures was done by re-docking the native ligand (onalespib, AT13387) into the active site of Hsp90. The results revealed the superposition of the redocked ligand and the co-crystallized ligand with a root mean square deviation of 2 Å. The results of the docking study of HAA_2020_ revealed a nice fitting into the ATPase site (Figure. 3), which could count for the inhibition in the catalytic activity of the ATPase. HA_A2020_ displayed a binding free energy (***ΔG_b_***) of −8.62 kcal/mol and an inhibition constant (***K_i_***) of 477.04 nM to Hsp90, compared with a binding free energy of −10.17 kcal/mol and an inhibition constant of 34.93 nM for onalespib. The 2/3D binding modes of HAA_2020_ and onalespib were generated using a Discovery Studio visualizer [35] and LigPlot^+^ (v.2.1) [36]. The LigPlot view showed the superposition of HAA_2020_ with the co-crystallized onalespib with similar binding interactions. HAA_2020_ showed four conventional hydrogen bonds with Asp93, Gly97 and Asp102 compared with two hydrogen bonds for onalespib. Moreover, HAA_2020_ exhibited diverse hydrophobic interactions with the hydrophobic residues in Hsp90 (Figure 3). 

### 2.3. MTT Combination Study

HAA_2020_, which showed good Hsp90 inhibition activity and fitted into the ATPase site of onalespib, was further selected to be tested in combination with a library of eleven compounds for the possible enhancement of its activity against HL60 cells. The eleven compounds represent the multiple-target inhibitor groups for leukemia. The maximum concentration for each of the eleven compounds used in the MTT cytotoxicity assay (HL60, 24 h) was 100 nM, and their resulting IC_50_ ranged between 7.9–56.5 nM, while the IC_50_ of HAA_2020_ was 1814 nM, which was similar to the IC_50_ of the same compound in a previous study and using the same cell line: 1710 nM [29]. Thus, the eleven compounds were considered for the combination with HAA_2020_ at 1:50, respectively. The IC_50_ of the eleven combinations were used with the IC_50_ of each drug alone including HAA_2020_, to calculate the combination index (CI) at Fa = 0.9, using the Compusyn software. The combination between HAA_2020_ and PHA-767491 resulted in a mild synergistic relationship (CI: 0.991), while the combination between HAA_2020_ and dinaciclib produced stronger synergism (CI: 0.628), and consequently enhanced the activity against HL60 cells to IC_50_ 4.9 nM (Table 2). The rest of the nine compounds resulted in antagonistic activity (CI: >1). The combination parameters for HAA_2020_ and dinaciclib are shown in Table 3. 

### 2.4. Cell Cycle Analysis

**Figure 4:**
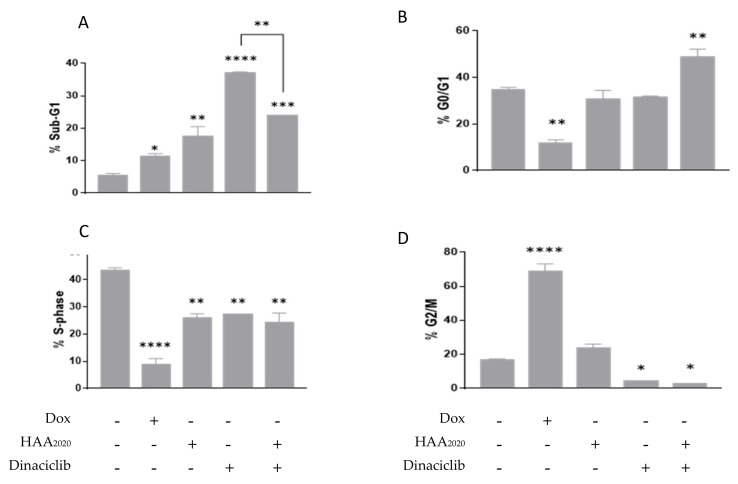
Cell cycle analysis in HL60 cells. Cells were treated (+) for 24 h with either 100 nM doxorubicin (Dox), 500 nM HAA_2020_, 10 nM dinaciclib or a combination of HAA_2020_ and dinaciclib (500 nM and 10 nM, respectively). Data are represented as mean ± SEM (*n* = 2, two independent experiments) for each of the phases of the cell cycle, sub-G_1_ (**A**), G_0_/G_1_ (**B**), S (**C**) and G_2_/M (**D**). Statistical differences, compared with the untreated control cells (-), were assessed by a one-way ANOVA with the Tukey’s post-hoc multiple comparison test (GraphPad Prism). *p* < 0.05 (*), *p* < 0.01 (**), *p* < 0.001 (***) and *p* < 0.0001 (****) were taken as significant.

The pan CDK inhibitor dinaciclib was selected as a candidate for further investigations with HAA_2020_ because it showed the best CI between the eleven inhibitors_._ The cell cycle distribution was used to examine the anti-proliferative effect of the HAA_2020_ and dinaciclib combination. Following a 24 h treatment, HAA_2020_ and dinaciclib (500 nM and 10 nM, respectively) elicited an increase in the sub-G_1_ phase in HL60 cells, and stronger than that observed for doxorubicin alone as a positive control (100 nM), with the highest increase attributed to dinaciclib. Moreover, the combination treatment showed a significant increase in the sub-G_1_ phase, but less than the effect caused by each of them alone (Figure 4A). Neither HAA_2020_ nor dinaciclib had an effect on the G_0_/G_1_ phase, however, their combination elicited a significant cell cycle arrest, as observed by an increase of cells in the G_0_/G_1_ phase (Figure 4B). However, this was not the case with the addition of doxorubicin, as an opposite observation was apparent, where G_0_/G_1_ was reduced. When comparing the differences in the S phase, all three compounds showed significant decreases compared with the untreated control, with the highest decrease attributed to doxorubicin, and little difference between either HAA_2020_, dinaciclib or their combination (Figure 4C). HAA_2020_ had no significant effect on the G_2_/M phase of the cell cycle, in contrast to dinaciclib, where G_2_/M was significantly reduced both alone or in combination with HAA_2020_ (Figure 4D). Furthermore, doxorubicin significantly increased cells in the G_2_/M phase. 

### 2.5. Detection of Apoptosis

**Figure 5:**
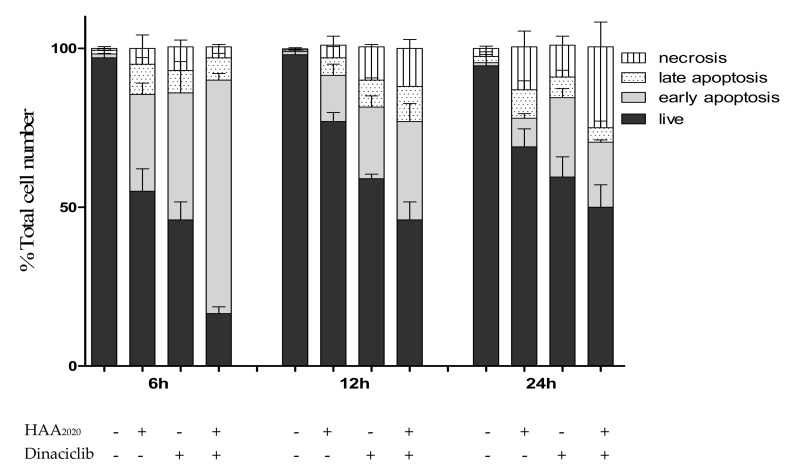
Detection of apoptosis in HL60 cells. Cells were treated for 6 h, 12 h and 24 h with either HAA_2020_ (500 nM), dinaciclib (10 nM) or their combination. Following treatment, the cells were stained with annexin V FITC/PI. A total of 20,000 single-cell events were acquired on a BC-500 flow cytometer and analyzed by the Expo 32 software. Data are represented as mean ± SEM (*n* = 3, two independent experiments) for each of the cell staining statuses: live cells (annexin V-/PI-), early apoptotic cells (annexin V+/PI-), late apoptotic cells (annexin V+/PI+) and necrotic cells (annexin V-/PI+).

The combination of HAA_2020_ and dinaciclib showed a synergistic G_0_/G_1_ arrest in HL60 cells following the 24 h treatment. Further, each of the two compounds induced significant apoptosis as shown in the sub-G_1_ phase, but together they induced less apoptosis compared with their effect alone. Thus, the annexin V FITC/PI assay was used at three time points including 24 h to investigate apoptosis in more detail. After 6 h of treatment, each of the two compounds caused an increase in the early apoptotic events compared with the control, and their combination caused synergistic early apoptosis compared with their effect alone in HL60 cells, all with minimal necrosis. At 12 h, each of the two compounds and their combination caused the same effect with a higher percentage of necrosis caused by dinaciclib and the combination, while after 24 h, the necrosis caused by the combination was equivalent to its early and late apoptotic events. The early apoptosis caused by the combination after 6 h, 12 h and 24 h was 70%, 30% and 20%, respectively, which may explain the superiority of HAA_2020_ and dinaciclib alone compared with their combination in producing a sub-G_1_ increase in HL60 cells after 24 h (Figure 5). Thus, this three-time point analysis showed that the most effective time point of the combination in HL60 cells is at 6 h. 

### 2.6. Real Time PCR

**Figure 6:**
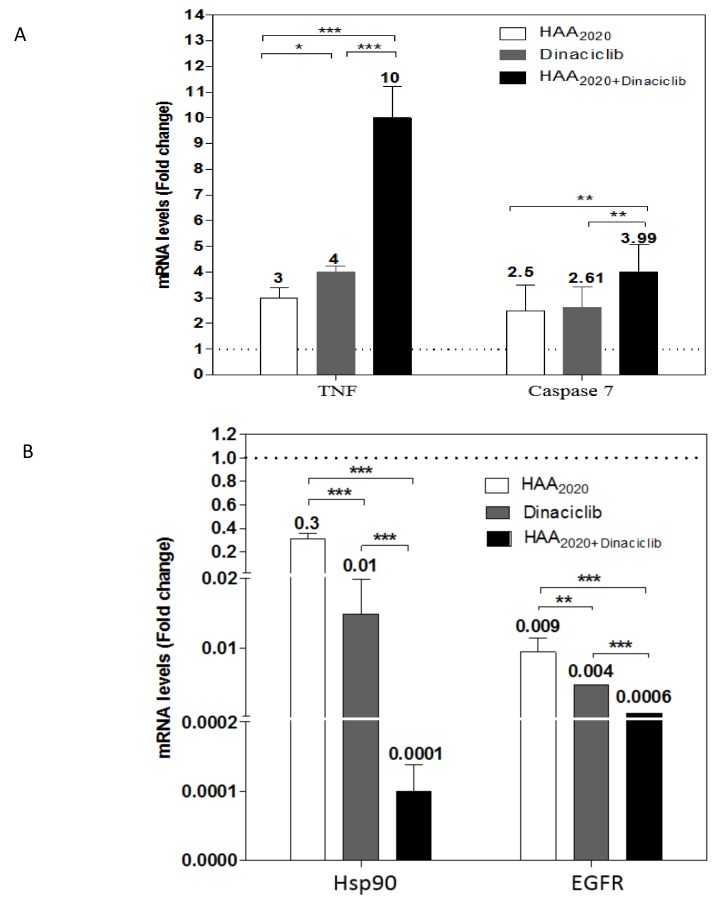
mRNA expression levels of TNF-α and caspase-7 (**A**), Hsp90-α and EGFR (**B**), compared with the control. HL60 cells were treated for 6 h with either HAA_2020_ (500 nM), dinaciclib (10 nM) or their combination. Data are represented as mean ± SEM (*n* = 3, two independent experiments). The results were expressed as fold-change compared with the untreated group. Statistical differences, compared with the untreated control cells (dashed line), were assessed by a one-way ANOVA with the Tukey’s post-hoc multiple comparison test. *p* < 0.05 (*), *p* < 0.01 (**) and *p* < 0.001 (***) were taken as significant.

For more details on the apoptotic process of the tested compounds, the mRNA amount of TNF and the caspase-7 genes were evaluated by real-time PCR, following the treatment of HL60 cells at 6 h with either HAA_2020_ (500 nM), dinaciclib (10 nM) or their combination. Each of the two compounds significantly upregulated the TNF-α and caspase-7 genes in addition to their combination, which showed a synergistic effect, suggesting involvement of both the extrinsic and intrinsic apoptotic pathways. A similar significant effect was exhibited against Hsp90α and its client protein EGFR, but in the decreasing mode, which indicates that the main activities of HAA_2020_ are maintained when it is combined to the CDK inhibitor dinaciclib (Figure 6).

### 2.7. Western Blotting

**Figure 7:**
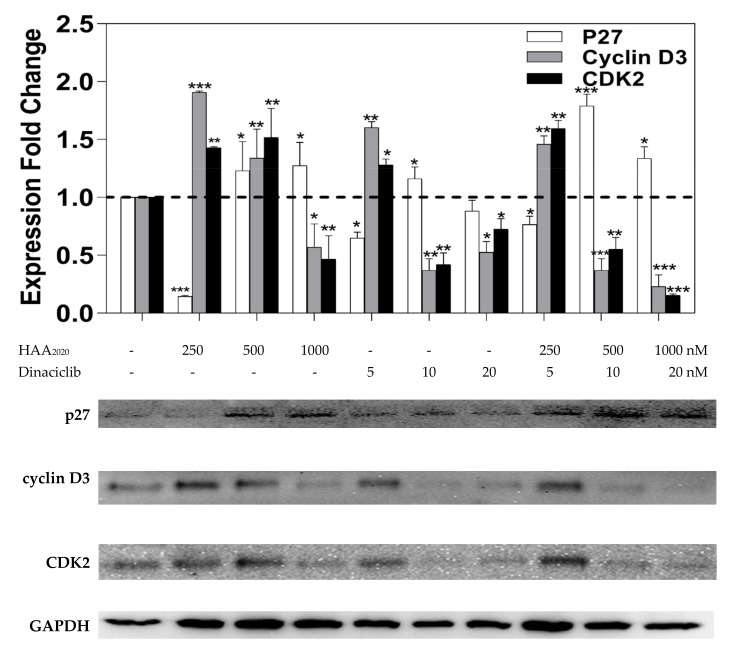
Expression of p27, cyclin D3 and CDK2 in HL60 cells treated with either HAA_2020_, dinaciclib or their combination as above for 6 h. Data are represented as mean ± SEM (*n* = 2, two independent experiments). The result was normalized as fold-change compared with the untreated group. Ratio above 1 (dashed line) shows the amount of mRNA for the three genes in the untreated cells (control = 1). Statistical differences, compared with the untreated control cells, were assessed by a one-way ANOVA with the Tukey’s post-hoc multiple comparison test. *p* < 0.05 (*), *p* < 0.01 (**) and *p* < 0.001 (***) were taken as significant. The Image J software was used for densitometry.

Western blotting was devised to study the effect of HAA_2020_ and dinaciclib (6 h) on the G_1_/S cell cycle check point regulatory proteins p27, cyclinD3 and CDK2. The HL60 cells were treated with HAA_2020_ (250 nM, 500 nM and 1000 nM), dinaciclib (5 nM, 10 nM and 20 nM) and their combination for 6 h. HAA_2020_ caused a dose-dependent increase in the p27 expression compared with the control GAPDH, while dinaciclib alone, or combined with HAA_2020_, caused an irregular increase in p27. Each of HAA_2020_, dinaciclib and their combinations caused a dose-dependent inhibition of cyclin D3 compared with the control, which was more pronounced by the combination. Most importantly, HAA_2020_ significantly reduced CDK2 at 1000 nM, while dinaciclib caused more inhibition at its IC_50_ (i.e., 10 nM). The combination of the two compounds caused a dose-dependent and significant decrease in CDK2 compared with GAPDH (Figure 7).

## 3. Discussion 

Many quinazolines which target EGFR and/or HER2, including afatinib, gefitinib, erlotinib and lapatinib, are established anticancer agents. HAA_2020_ (4-(2-(4-Oxo-2-thioxo-1,4-dihydroquinazolin-3(2*H*)-yl)ethyl)benzene sulfonamide) is a previously synthetized quinazoline, which showed cytotoxicity against the HL60 and K562 leukemia cell lines and spared the MRC5 normal cells. HAA_2020_ was two folds stronger against HL60 cells, compared with K562 cells (IC_50_: 1.71 µM and 3.67 µM, respectively). It also displayed a potent inhibitory activity against the VEGFR2, EGFR and HER2 kinases [29]. 

In the search for new anticancer agents, Hsp90 poses an attractive target in AML, as it is involved in the regulation of survival, cell proliferation and apoptosis [13,14]. Hsp90 has many important client proteins including the VEGFR2, EGFR and HER2 kinases [15]. A number of previously reported quinazolines have shown Hsp90 inhibition activities including 3-phenyl-2-styryl-3H-quinazolin-4-one [37]. The SPR-based binding assay results in this study showed the efficient interaction of HAA_2020_ with the Hsp90 protein at 51 nM KD, which was greater towards the chaperone compared with that determined for 17-AAG. This result was supported by a molecular docking study, which showed that HAA_2020_ fitted nicely into the ATPase site, which could possibly be due to its catalytic activity. Thus, HAA_2020_ can be considered as a novel Hsp90 inhibitor, among its previously described activities. In a previous study, the treatment of HL60 cells with 17-AAG inhibited Hsp90 [38].

With the purpose of enhancing the activity against AML, which is represented in this study by HL60 cells, HAA_2020_ was combined with each of the eleven anti-leukemic inhibitors for 24 h. Only two inhibitors resulted in a synergistic effect with HAA_2020_: PHA-767491 and dinaciclib, both CDK inhibitors. However, dinaciclib showed more synergism with HAA_2020_ (CI: 0.62) compared with PHA-767491 (CI: 0.99), which was comparable with the synergistic activity of the ganetespib and cytarabine combination (CI: 0.47 [39]). As expected, the combination of HAA_2020_ and dinaciclib enhanced the cytotoxicity against HL60 (IC_50_: 4.9 nM) compared with the IC_50_ of HAA_2020_ and dinaciclib alone (1814 nM and 8.2 nM, respectively). CDK inhibitors alone have previously shown promising results in the clinic for AML, including alvocidib and dinaciclib [10,11,12]. Moreover, in combination, the CDK inhibitor (SCH727965) and the Hsp90 inhibitor (NVP-AUY922) were found to induce apoptosis and cell cycle disruption in sarcoma cells, while it spared the normal osteoblasts [27]. In another combination study, the resistance in melanoma patients following the use of the Hsp90 inhibitor XL888 with the BRAF inhibitor vemurafenib was reverted by the use of dinaciclib as a CDK2 inhibitor [22]. Thus, dinaciclib was selected for further mechanistic investigations with HAA_2020_. 

Following the 24 h treatment of HL60 cells, the combination of dinaciclib and HAA_2020_ elicited a significant G_0_/G_1_ cell cycle arrest. However, each of the two compounds showed a stronger increase in the sub-G_1_ phase compared with doxorubicin and with their combination. Thus, a three-time point, more apoptosis-specific assay was performed to investigate the reason behind the fall of the combination to achieve a higher sub-G_1_ effect compared with HAA_2020_ and dinaciclib alone. The annexin V FITC/PI assay showed that the most effective early and late apoptosis inducing time point for the combination was at 6 h compared with 12 h and 24 h (combined early and late apoptosis at the three time points: 80%, 45% and 25%, respectively). All of this occurred parallel with the time-dependent increasing necrosis in the HL60 population (necrosis at the three time points: 4%, 12% and 22%, respectively). Similarly, 17-AAG induced 80% apoptosis in HL60 cells at 1 µM (48 h), which was assessed by the annexin V assay, and it caused an increase in the sub-G_1_ HL60 cells from 3.5% in the untreated cells to 13% at 1 µM, simultaneously with a G_0_/G_1_ arrest of 52% (compared with 34% in the untreated cells) at 6 h, all at the expense of reduced S cell cycle phase events (from 52% in the untreated cells down to 7% in the treated ones) [38]. Further, in another previous report, dinaciclib alone increased the sub-G_1_ phase at 4–16 nM in HL60 cells, and induced high apoptosis following 2 h of treatment, which decreased by two and five folds following 6 h and 24 h, respectively [12].

Previous reports have shown that the stimulation of TNF-α results in the upregulation of caspases 3/7, which leads to apoptosis in various cancer cells [40]. Moreover, Hsp90 is regarded as an anti-apoptotic protein that can be downregulated by the stimulation of TNF-α [41]. In this regard, 17-AAG was previously shown to increase caspases 3/7/9 in HL60 and mesothelioma cell lines [24,38]. Thus, to confirm whether the induced apoptosis in HL60 cells involves this pathway or not, HAA_2020_ and dinaciclib and their combinations were tested at 6 h, resulting in the significant simultaneous upregulation of TNF-α and caspase-7, confirming the involvement of these multiple apoptotic pathways in the effect of the combination. The combination was also confirmed to show the simultaneous and significant inhibition of Hsp90 and its client protein EGFR in HL60 cells at 6 h, which indicates that the main activities of HAA_2020_ are maintained when it is combined with the CDK inhibitor. Similarly, AT13387, a drug candidate in clinical trials, showed a dual EGFR and Hsp90 inhibition mechanism of action [42,43]. 

In cell cycles, the G_1_/S phases are modulated by the CDK2, CDK4/6 and cyclins A,D checkpoint proteins, while the G_2_/M phases are modulated by the CDK1 and cyclin B check points [44]. The cell cycle effect of HAA_2020_ and dinaciclib was tested at 24 h, revealing an increase in the sub-G_1_ and G_0_/G_1_ phases. To confirm the cell cycle effect of the combination at 6 h, which showed more apoptosis in HL60, p27, CDK2 and cyclin D3 were tested. It was found that our combination downregulated p27, cyclin D3 and CDK2, which agrees with previous reports showing that the decrease in Hsp90 is associated with the G_1_ arrest in leukemia cells [16,21]. Additionally, XL888 synergizes with dinaciclib to inhibit CDK2 [22]. Further, the Hsp90 inhibitor 17-AAG was found to increase p27 and caspases 3/7 in mesothelioma cell lines [24]. 

Hsp90 inhibitors are important assets in the anticancer armory, as they actively help the control of steroidal hormone receptors and transcription factors, in addition to many kinase pathways in our bodies. In the preclinical settings, Hsp90 inhibitors showed more successful stories compared with the clinical studies. Ganetespib, for example, showed a suppression of the AML-patients’ derived blast cells alone or when combined with cytarabine (CI = 0.47). That activity was mediated by the downregulation of Akt and the induction of apoptosis in HL60 cells [39]. There are currently many Hsp90 inhibitors undergoing clinical trials: , 2 and 1-2. These inhibitors include, among others, SNX-5422 for chronic lymphocytic leukemia (combined with ibrutinib, phase 1) and AT13387 for EGFR-mutant lung cancer (combined with erlotinib hydrochloride, phase 1–2). Moreover, the Hsp90 inhibitors 17-AAG and 17-DMAG showed CDK4 inhibition effects, alone or combined with trastuzumab or sorafineb in separate clinical trials. 17-AAG also exhibited a CDK4 and cyclinD1 inhibition when combined with irinotican. In another clinical trial, AT13387 inhibited CDK4 and caspase-3, while BIIB021 inhibited both CDK4 and HER2 [42,43].

**Figure 8:**
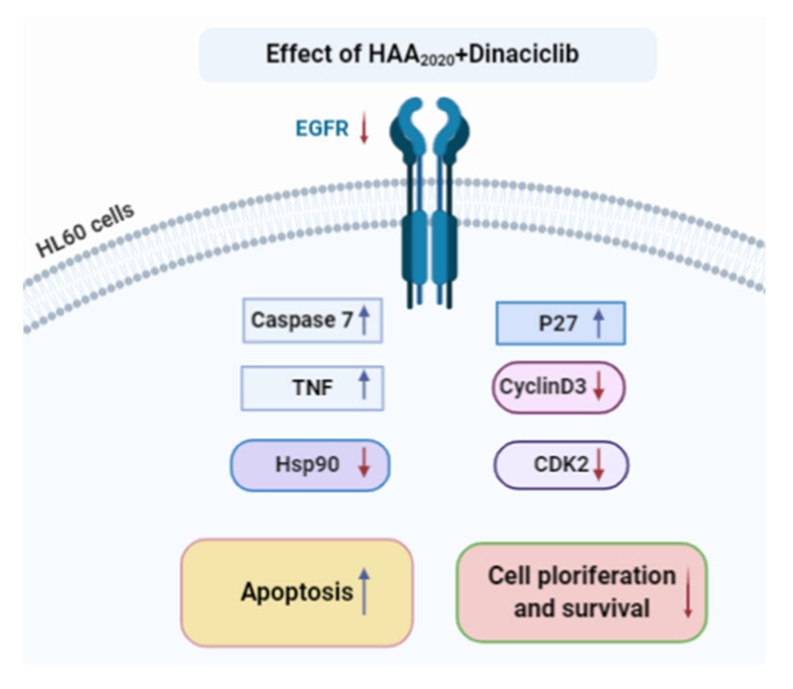
The effect of the HAA_2020_ and dinaciclib combination on apoptosis, cell proliferation and survival of HL60 cells. Created in BioRender.com.

Taking all that together, breast and lung cancers are the only up-to-date clinically successful target organs for Hsp90 inhibitors. Thus, there is a continuous need for mechanistic and combination studies in other cancer platforms, including leukemia, which could help in understanding the role of Hsp90 inhibitors in the clinical stage, and to overcome the efficacy and side effects issues [42,43]. The number of drugs available for leukemia, including AML, are hindered by an array of resistance mechanisms, which necessitates the readiness of more efficient drugs on the front line. The results of this study demonstrate the efficiency of HAA_2020_ as a Hsp90 inhibitor, which also induced apoptosis and cell cycle effects in HL60 cells, alone or when combined with the pan CDK inhibitor dinaciclib. The combination activity was associated with increased caspase-7 and TNF-α, leading to apoptosis in HL60 cells. In addition, it upregulated p27 simultaneously with the inhibition of cyclinD3 and CDK2, leading to abolished HL60 proliferation and survival (Figure 8). The combination of Hsp90 and CDK inhibitors could pave the way for important emerging drugs in the clinic. Future work may involve in vivo testing of HAA_2020_, alone or with dinaciclib or other CDK inhibitors, and comparing the activity with AML-gold standard therapies like cytarabine, all of which could better the understanding of the relationship between Hsp90 and CDK inhibition in AML. 

## 4. Materials and Methods

### 4.1. Compounds and Reagents

PHA-767491, dinaciclib, ON123300, AMG925, KW2449, AZD2932, dasatinib, BGJ398, saracatinib, lapatinib and taselisib were purchased from Selleckchem, Houston, TX, USA. Doxorubicin was purchased from Cayman, Ann Arbor, MI, USA. All reagents and kits, including ethanol and dimethyl sulfoxide (DMSO), were purchased from Sigma Chemical Co. Jeddah, MK, KSA, unless otherwise reported.

### 4.2. Methods

#### 4.2.1. Surface Plasmon Resonance

Surface plasmon resonance (SPR), which is an optical analytic technique for measuring the kinetic and thermodynamic parameters of ligand−protein complexes’ formation and affinity, is widely used to investigate enzyme/inhibitor interactions [30,31,32]. Recently this approach was successfully applied to study the binding of small molecules to Hsp90 [33,45,46,47]. SPR experiments were performed in this study as described elsewhere [33]. Briefly, analyses were carried out using a Biacore 3000 optical biosensor equipped with research-grade CM5 sensor chips (GE Healthcare, Piscataway, NJ, USA). Two separate recombinant Hsp90 surfaces, a BSA surface and an unmodified reference surface, were prepared for simultaneous analyses. The recombinant human Hsp90α (SPP-776, Stress-gen Bioreagents Corporation, Victoria, anada) was dissolved at 100 μg/mL in 10 mM sodium acetate, pH 5.0, then it was immobilized on individual sensor chip surfaces at a flow rate of 5 μL/min using standard amine-coupling protocols to obtain densities of 8−12 kRU. Compounds **1**, **4** and **5**, [29], as well as 17-AAG used as the positive control [48,49], were dissolved in 100% DMSO to obtain 4 mM solutions and diluted 1:1000 (*v*/*v*) in PBS (10 mM NaH_2_PO_4_, 150 mM NaCl, pH 7.4) to a final DMSO concentration of 0.1%. A series of concentrations were prepared as 2-fold dilutions into a running buffer: for each sample, the complete binding study was performed using a six-point concentration series, typically spanning 0.025−4 μM, and triplicate aliquots of each test compound were dispensed into single-use vials. Multiple blank samples of the running buffer alone were included in each analysis. Binding experiments were performed at 25 °C, using a flow rate of 50 μL/min, with 60 s monitoring of association and 250 s monitoring of dissociation. Simple interactions were adequately fit to a single-site bimolecular interaction model (A + B = AB), yielding a single K_D_. Sensorgram elaborations were performed using the BIAevaluation software provided by GE Healthcare (Data files for SPR are available from the Supplementary Material S3, and can be opened by dragging each of the files into https://filext.com/file-extension/BLE).

#### 4.2.2. Molecular Docking Study

A comparative molecular docking study was performed for the best Hsp90 inhibiting compound from the previous analysis, **1** (4-(2-(4-Oxo-2-thioxo-1,4-dihydroquinazolin-3(2*H*)-yl)ethyl)benzene sulfonamide, named HAA_2020_ hereafter) and onalespib (AT13387, Figure 2) into the active site of Hsp90. The binding modes, affinities and the binding free energies of HAA_2020_ were evaluated and compared with those of the co-crystallized ligand (onalespib). The crystal structure of the Hsp90 (pdb code: 2XJX) co-crystallized with onalespib (AT13387) was obtained from Protein Data Bank (http://www.rcsb.org/pdb) in an X-ray resolution of 1.66 Å [18]. Preparations of the protein and ligand files, and the energy minimization of HAA_2020_ and onalespib were done following the previous report [50]. In addition, the preparation of the grid parameter files was done using AutoGrid according to the same previous report [50]. The 3D grid dimensions were set to the default values with 0.375 Å spacing. The protein structure was set as a rigid file, while HAA_2020_ and onalespib were docked as flexible molecules. The genetic algorithm was used as the search parameter, and docking parameters were set to the default values. The docking poses were scored and ranked by AutoDock in a decreasing order of their binding free energy. The top ten conformations of the protein–ligand complexes were clustered. The binding modes of the best fit conformations of HAA_2020_ and onalespib were analyzed. Different types of ligand–protein interactions were vitalized using a Discovery Studio visualizer and LigPlot^+^. The 2/3D binding modes were generated showing both the hydrogen bonding and hydrophobic interactions (Figure 3). 

#### 4.2.3. Cell Culture

HL60 suspension cells (Hematopoietic neoplasm, Human acute myeloid leukemia: AML, Myc+, CLL1, CD33, CD123 and CD135) were obtained from the ATCC. Cells were maintained in RPMI-1640 media (10% FBS, 1% Antibiotic-Antimycotic, Gibco) at 100% humidity, 37 °C and 5% CO_2_. To keep logarithmic growth, a maximum of 5 × 10^5^ cells/mL were sub-cultured (1–10 passages).

#### 4.2.4. MTT Combination Study

The cytotoxicity of HAA_2020_ and each of the eleven inhibitors library was assessed by an MTT assay, as previously reported [51,52]. The eleven inhibitors were selected depending on the multiple existing and putative leukemia targets, mainly FLT3, PDGFR, Bcr-Abl, EGFR and CDKs (Table 2) [53,54]. HL60 cells were cultured in 96-well (5 × 10^4^/well). The final HAA_2020_ concentrations were 0, 312.5, 625, 1250, 2500 and 5000 nM (based on IC_50_ obtained from previous report: [29]), while the final concentrations of the eleven inhibitors were 0, 6.25, 12.5, 25, 50 and 100 nM (based on IC_50_ obtained from Sellechem.com). The final DMSO concentration was 0.1%, (*n* = 3). Depending on the average IC_50_ of HAA_2020_ and the eleven inhibitors in HL60 cells (Table 2), the combinations of HAA_2020_ and each of the eleven inhibitors were performed in a 50:1 ratio. Plates were incubated for 24 h, followed by the addition of MTT (3 h, Life technologies) and centrifugation of the 96-well plates at 400 g/5 min (Eppendorf 5430), resulting in the precipitation of cells at the bottom of the plates. That was followed by suction of the media and the addition of DMSO to each well. The absorbance was read on a multi-plate reader (BIORAD, PR 4100). The optical density of the purple formazan A_550_ is proportional to the number of viable cells. The compound concentration causing 50% inhibition (IC_50_) compared with the control cell growth (100%) was determined using GraphPad Prism. The CompuSyn software was used to calculate the combination index (CI) [(D)_1_/(Dx)_1_]+ [(D)_2_/(Dx)_2_], where (D)_1_ and (D)_2_ are concentrations of drug A and drug B, which are employed in the combination study to calculate the IC_50_. (Dx)1 and (Dx)2 are the IC_50_ of single drugs A and B. CI <1: synergism, CI = 1: additivity and CI >1: antagonism [55].

#### 4.2.5. Cell Cycle Analysis 

Cell cycles are series of changes resulting from the multiple checkpoints and occurring from the initial phase of the cell formation leading to its division. The disruption of the cell cycle causes cancer, which usually takes place in the G_1_/S (modulated by CDK4/6-cyclinD and CDK2-cyclinA) or the G_2_/M (modulated by CDK1-cyclinB) check points [44]. Following the treatment of HL60 cells (5 × 10^5^) with each of HAA_2020_, dinaciclib and their combination (500 nM + 10 nM, respectively) for 24 h, the cells were fixed in 70% ethanol and then processed for a cell cycle analysis using propidium iodide (PI, Santa Cruz, CA, USA), as previously described [56]. A total of 20,000 single-cell events were acquired on a flow cytometer (NovoCyte 3000, ACEA biosciences, San Diego, CA, USA) and analyzed using the cell cycle algorithm of the NovoExpress software (version 1.4.1 ACEA biosciences, San Diego, CA, USA).

#### 4.2.6. Detection of Apoptosis

As a major cell death route, apoptosis is regarded as a non-immunogenic, energy-dependent selective programmed pathway, which cleans dead cells. Apoptosis occur either extrinsically (in the cytoplasm) via the Fas death receptor, a member of tumor necrosis factor (TNF) receptor family, or intrinsically (in the mitochondria), which leads to the release of the cytochrome-c, thus activating cell death signals. Both of the pathways lead to the activation of certain proteases called caspases, ending with cell death [57,58]. In this study, apoptosis was quantified by detecting the cell surface exposure of phosphatidylserine (PS) in apoptotic cells using annexin V FITC/PI, following a previous report [59]. HL60 cells were seeded in 6-well plates at 5 × 10^5^ cells/well for overnight, before the treatment with HAA_2020_, dinaciclib and their combination (500 nM and 10 nM, respectively) for 6 h, 12 h and 24 h. Cells were inspected microscopically before and after treatment to observe the morphological changes. Then they were collected, centrifuged and the pellets were re-suspended in a binding buffer. Annexin V FITC (Invitrogen, Waltham, MA, USA) was then added to each sample and incubated at room temperature in the dark. A further binding buffer and PI were added. Samples were analyzed by flow cytometry (BC 500, Miami, FL, USA) within 1 h. Viable cells were differentiated from early and late apoptotic/necrotic cells by annexin V (x axis) and PI staining (y axis).

#### 4.2.7. Gene Expression Analysis and Quantitative Real Time PCR

The total RNA resulting from the stimulation of HL60 cells with HAA_2020_, dinaciclib and their combination was isolated using an RNA isolation mini kit (Thermo Fisher Scientific, Waltham, MA, USA) according to manufacturer’s instructions. The NanoDrop 2000 spectrophotometer (Thermo Scientific, Waltham, MA, USA) was used to determine the concentration and purity of the isolated RNA. The purity of the isolated RNA and its integrity was validated using 1% agarose gel electrophoresis. Subsequently, the complementary DNA (cDNA) was synthesized using the isolated RNA (2 µg) based on the instructions of the manufacturer, using the RevertAid First Strand cDNA Synthesis Kit (Thermo Fisher Scientific, Waltham, MA, USA). The RT-PCR was carried out in a 96-well plate by the RT-PCR platform using the Applied Biosystems 7500 Fast Real Time PCR System, following a previous report [60]. Briefly, HL60 cells were seeded in 6-well plates (1 × 10^6^ cells/well) and incubated overnight, before treatment with HAA_2020_, dinaciclib and their combination (500 nM and 10 nM, respectively) for 6 h. The RT-PCR mixture contained 10 µL of 2X SYBR Green I Master mix, 10 ng of cDNA, 0.4 µM of each human primers (TNF-α, caspase-7, Hsp-90α, EGFR and GAPDH as the housekeeping gene, (Table 4), Applied-Biosystems, Thermo Fisher Scientific, Waltham, MA, USA) and PCR-grade water up to a total volume of 20 µL. The RT- PCR program consisted of 45 cycles of denaturation at 95 °C for 15 s followed by annealing/extension at 60 °C for 60 s. All RT-PCR reactions were applied in triplicates and repeated 3 times and included a negative control (no template). Standard comparative methods (ΔCt) were used to evaluate the genes’ expressions. The raw Ct values were converted into relative expression levels (fold-change) using the formula 2^−∆∆Ct^.

#### 4.2.8. Western Blotting

To confirm the cell cycle effect, Western blotting was used to investigate the expression change of the cell cycle proteins p27, cyclinD3 and CDK2. HL60 cells (1 × 10^6^ cells/well of 6-well plates) were treated with HAA_2020_ (250 nM, 500 nM and 1000 nM), dinaciclib (5 nM, 10 nM and 20 nM) and their combination for 6 h (Figure.7). The total proteins were isolated after the complete lysis of cells by a lysis buffer. The concentration of the total protein was determined by the Bradford method. The loading protein samples were electrophoresed on a polyacrylamide gel and transferred to a membrane. The membrane was incubated with p27, cyclinD3 and CDK2 antibodies (Cell signalling) for 2 h at room temperature and the secondary antibody GAPDH for 1 h at room temperature. The immunoreactivity was visualized by chemiluminescence using horseradish peroxidase (HRP)-conjugated secondary antibodies, and their image was detected by a scanner (GeneGenome, Syngene BioImaging) [61].

### 4.3. Statistics

Statistical differences of samples, compared with the untreated control cells, were assessed by a one-way ANOVA with the Tukey’s post-hoc multiple comparison test (GraphPad Prism Version 5). *p* < 0.05 (*), *p* < 0.01 (**), *p* < 0.001 (***) and *p* < 0.0001 (****) were taken as significant. 

## Figures and Tables

**Table 1:** Thermodynamic constants measured by surface plasmon resonance (SPR) for the interaction between the tested compounds and immobilized Hsp90α.

Compound	K_D_ (nM)^a^
1^b^	51.0 ± 2.9
4	NB^c^
5	NB
17-AAG	360.0 ± 21.9

^a^: Results were given as the mean ± standard deviation; ^b^: named HAA_2020_ in this study; ^c^: No binding.

**Table 2:** Cytotoxic IC_50_ values of HAA_2020_, the eleven inhibitors and their combinations in HL60 cells (24 h, nM).

No.	Inhibitor	Targets	IC_50_ nM	IC_50_ (nM) Combination HAA_2020_/Inhibitor ^a^ (50:1)	CI ^b^ at Fa = 0.9
1	PHA-767491	CDK1/2,CDK5, Cdc7/CDK9, GSK3-β,MK2 PLK1, CHK2	26.1 ± 3.8	21.8 ± 3.2	0.991
2	Dinaciclib	CDK1, CDK2, CDK5, CDK9	8.2 ± 0.3	4.9 ± 1.3	0.628
3	ON123300	CDK4, Ark5, PDGFRβ, FGFR RET, Fyn	37.3 ± 3.5	1872.5 ± 45.9	>1
4	AMG 925	CDK4/FLT3	54.4 ± 7.7	3355 ± 347.8	>1
5	KW2449	FLT3, EGFR, FGFR1, Bcr-Abl PDGFRβ, IGF-1R	56.5 ± 10.8	2535.5 ± 318.9	>1
6	AZD2932	FLT3, c-Kit, VEGFR, PDGFRβ	15.2 ± 3.1	2394 ± 380.4	>1
7	Dasatinib	c-Kit, Abl, Src	12.1 ± 2.9	3036 ± 181.1	>1
8	BGJ398	Kit, FGFR, VEGFR2, Abl, Fyn, Lck, Lyn, Yes	16.2 ± 1.7	2952.5 ± 170.6	>1
9	Saracatinib	EGFR, Src, Yes, Fyn, Lyn, Blk, Fgr, Lck, Abl	16.8 ± 1.2	1821.5 ± 40.3	>1
10	Lapatinib	EGFR, HER2	12.1 ± 2.0	2383.5 ± 245.3	>1
11	Taselisib	PI3Kα/δ/γ	7.9 ± 0.4	2647 ± 203.6	>1
	**HAA**_**2020**_	VEGFR2, EGFR, HER2	1814.5 ± 230.8	-	-

^a^: inhibitors 1–11. ^b^: Combination index. (-): not applicable. Data are represented as mean ± SD (*n* = 3). Experiment was repeated 3×. IC_50_ of some combinations is more than 100 nM because the IC_50_ of HAA_2020_ alone is more than 100 nM.

**Table 3:** The combination index parameters of HAA_2020_, dinaciclib and their combination.

Drug/Combination (1:50)	Dm	m	r	CI ^a^ at Fa = 0.9
Dinaciclib	15.985	–0.358	–0.911	-
HAA_2020_	0.340	–1.184	–0.997	-
HAA_2020_ + Dinaciclib	0.552	–0.722	–0.998	0.628

^a^: Combination index. Definition of Dm, m and r values are found in the Supplementary Material (S2).

**Table 4:** Sequence of Hsp90α, TNF-α, caspase-7, EGFR and GAPDH primers.

Gene	Sequence
Hep90α	(F) TTGGTTACTTCCCCGTGCTG (R) GCCTTTTGCCGTAGGGTTTC
TNF-α	(F) CTCTTCTGCCTGCTGCACTTTG (R) ATGGGCTACAGGCTTGTCACTC
caspase-7	(F) GGACCGAGTGCCCACTTATC (R) TCGCTTTGTCGAAGTTCTTGTT
EGFR	(F) GCGTCTCTTGCCGGAATGT (R) GGCTCACCCTCCAGAAGGTT
GAPDH	(F) AGGTCGGTGTGAACGGATTTG (R) TGTAGACCATGTAGTTGAGGTCA

## Supplementary Materials

The following are available online at https://www.mdpi.com/1420-3049/25/9/2220/s1, Table S1: Structure of compounds 1–5; S2: Definition of Dm, m and r values; Data files S3: Original data files for SPR.

## Abbreviations

17-AAG	17-N-allylamino-17-demethoxygeldanamycin
AML	Acute Myeloid Leukaemia
CDK	Cyclin dependent kinases
CI	Combination index
EGFR	Epidermal growth factor receptor
Hsp90	Heat shock protein 90
KSA	Kingdom of Saudi Arabia
M1	AML without maturation
PI	Propidium iodide
SPR	Surface Plasmon Resonance
TNF-α	tumor necrosis factor alfa
VEGF	Vascular endothelial growth factor
VEGFR	Vascular endothelial growth factor receptor
