# Supplementary material for: Synergistic Anti Leukemia Effect of a Novel Hsp90 and a Pan Cyclin Dependent Kinase Inhibitors
Source: Molecules. 2020 May 8;25(9):2220. doi: 10.3390/molecules25092220 (PMC7248782; doi:10.3390/molecules25092220)
Supplement: Supplementary file 1 [file molecules-25-02220-s001.zip › molecules-789980-supplememtary materials-proof/Supplementary materials.docx]

**Supplementary material**

**Table S1.** Structure of compounds 1–5

| Compound No. | Structure | |
| --- | --- | --- |
| **1** | 4-(2-(4-Oxo-2-thioxo-1,4-dihydroquinazolin-3(2*H*)-yl)ethyl)benzenesulfonamide |  |
| **2** | 2-((4-Oxo-3-(4-sulfamoylphenethyl)-3,4-dihydroquinazolin-2-yl)thio)acetamide |  |
| **3** | 2-((4-Oxo-3-(4-sulfamoylphenethyl)-3,4-dihydroquinazolin-2-yl)thio)-*N*-phenylacetamide |  |
| **4** | *N*-(4-Bromophenyl)-2-((4-oxo-3-(4-sulfamoylphenethyl)-3,4-dihydroquinazolin-2-yl)thio)acetamide |  |
| **5** | *N*-(4-Chlorophenyl)-2-((4-oxo-3-(4-sulfamoylphenethyl)-3,4-dihydroquinazolin-2-yl)thio)acetamide |  |

**S2.** Definition of Dm, m and r values (obtained from www.combosyn.com/rptExamp.html):

**Dm:** The median effect dose, in this case it is IC_50_ value, which indicates “potency”. The value can be obtained from the X-intercept of the ME-plot.

**m:** The “slope”of the median-effect (ME) plot, the dynamic order, or the “shape” of dose-effect curve; m = 1, >1 and

**r** : The linear correlation coefficient of the ME-plot. It signifies the “conformity” of the data with the mass-action law; an indication of how good the data are, when r = 1, it is perfect; For in vitro experiment, usually r > 0.95 are considered good or acceptable.

**Data files S3.** Original data files for SPR

| File* No. | Description |
| --- | --- |
| 1 | Compound 1 |
| 2 | Compound 4 |
| 3 | Compound 5 |
| 4 | 17-AAG |

*Files can be opened by dragging each of them into <https://filext.com/file-extension/BLE>.

Link to the 4 files:

<https://1drv.ms/u/s!AuA2qju9wwwolErkDht9gKx4XWpb?e=FHP14H>
